# Comparing three short questionnaires to detect psychosocial dysfunction among primary school children: a randomized method

**DOI:** 10.1186/1471-2458-9-489

**Published:** 2009-12-28

**Authors:** Antonius GC Vogels, Matty R Crone, Femke Hoekstra, Sijmen A Reijneveld

**Affiliations:** 1 Prevention and Healthcare, TNO (Netherlands Organisation of Applied Scientific Research) Quality of Life, PO Box 2215, 2301 CE Leiden, The Netherlands; 2 Department of Public Health and Primary Care, Leiden University Medical Center, Leiden, The Netherlands; 3 Epidemiology, Documentation and Health Promotion, Municipal Health Service Amsterdam, Amsterdam, The Netherlands; 4 Department of Health Sciences, University Medical Center Groningen, University of Groningen, Groningen, The Netherlands

## Abstract

**Background:**

Good questionnaires are essential to support the early identification of children with psychosocial dysfunction in community based settings. Our aim was to assess which of three short questionnaires was most suitable for this identification among school-aged children

**Methods:**

A community-based sample of 2,066 parents of children aged 7-12 years (85% of those eligible) filled out the Child Behavior Checklist (CBCL) and - randomly determined - one of three questionnaires to be compared: the Strengths and Difficulties Questionnaire with Impact Supplement (SDQ), the Pediatric Symptom Checklist (PSC) and the PSYBOBA, a Dutch-origin questionnaire. Preventive Child Healthcare professionals assessed children's psychosocial functioning during routine health examinations. We assessed the scale structure (by means of Structural Equation Modelling), validity (correlation coefficients, sensitivity and specificity) and usability (ratings by parents and professionals) of each questionnaire and the degree to which they could improve the identification based only on clinical assessment (logistic regression).

**Results:**

For the three questionnaires, Cronbach's alphas varied between 0.80 and 0.89. Sensitivities for a clinical CBCL at a cut off point with specificity = 0.90 varied between 0.78 and 0.86 for the three questionnaires. Areas under the Receiver Operating Curve, using the CBCL as criterion, varied between 0.93 and 0.96. No differences were statistically significant. All three questionnaires added information to the clinical assessment. Odds ratios (95% confidence intervals) for added information were PSC: 29.3 (14.4-59.8), SDQ: 55.0 (23.1-131.2) and PSYBOBA: 68.5 (28.3-165.6). Parents preferred the SDQ and PSYBOBA. Preventive Child Health Care professionals preferred the SDQ.

**Conclusions:**

This randomized comparison of three questionnaires shows that each of the three questionnaires can improve the detection of psychosocial dysfunction among children substantially.

## Background

Psychosocial problems are quite common among children. Prevalence rates vary with age, methods used and sample. Brugman et al. reported that clinicians identified such problems among 25% of 4480 school aged children invited for a routine health check-up[1]. In another study[2], family physicians identified problems in approximately 22% of 898 children aged 5 to 15 years. Kelleher and co-workers reported that paediatric and family practice clinicians identified psychosocial problems among 19% of a national sample of children aged 4-15 years[3]. Reijneveld et al found that nearly nine percent of parents reported frequent concerns, mostly about their child's behaviour[4]. Only a minority of children with such problems are treated for these problems. Verhulst found that only 13% of children with behavioural or emotional problems were referred to mental health care[5]. Reliable and valid questionnaires can improve early detection of such problems and the following treatment of these children and thereby improve these children's prognosis significantly and substantively[6].

Community paediatric services, like those in the USA and the Netherlands, offering routine health care services to the population as a whole, are in a unique position to detect children with psychosocial problems. In the Netherlands this early detection is an explicitly formulated task of the existing Preventive Child Healthcare (PCH). Dutch PCH services invite all children in their region for a regular health examination at specified moments in their development. This health examination includes both physical and mental health. When problems are detected children are referred to the family doctor or specialized care. The level of show up is high: PCH reaches about 95% of all children. Without reliable and valid questionnaires, many children with mental health problems are likely to be missed. For instance, Brugman and co-workers showed that PCH, without such instruments, identified problems among 25% of the children, but 43% of the children with a clinical score on the CBCL were missed[1]. Introducing valid instruments will also reduce the number of false positives.

The CBCL is a strongly validated instrument to assess psychosocial problems in children. Unfortunately, it does not meet the practical requirements for widespread use in Dutch PCH. Instruments to be used in PCH must be short, easy to answer and - due to the limited time available for a standard health examination - easy to score. Clearly, the CBCL with its 120 items does not meet this criterion.

Several short instruments are available for use among school-aged children that do meet these practical requirements: the Pediatric Symptom Checklist (PSC) [7-10], the Strengths and Difficulties Questionnaire (SDQ)[11,12], and a newly developed Dutch instrument, the PSYBOBA (a Dutch acronym, standing for 'Questionnaire for psychosocial problems among primary school children aged 7 to 12)[13]. These questionnaires aim to detect behavioural and emotional problems in children in primary education and are available in parent form.

The aim of this study was to compare the psychometric properties of these questionnaires in a randomized diagnostic method to select the best questionnaire for routine use in community based PCH for primary school children. More specifically: which questionnaire enables PCH to distinguish best between children with and without problems, offers PCH most information not already available from other sources and is most suitable for use in routine practice, for parents and for PCH professionals?

## Methods

We developed a randomized procedure in which we compared the questionnaires on a number of predefined criteria, using data that we collected in an identical way for each of the questionnaires to be assessed. To guarantee complete equivalence of data, we used a community sample of parents who all filled out the Child Behavior Checklist (CBCL), but were randomized to fill out one of the three questionnaires to be evaluated (PSC, SDQ or PSYBOBA). Next, we obtained information from the PCH professionals, blinded for the parental questionnaire, about background characteristics and psychosocial problems detected by PCH professionals.

### Sample

We obtained our sample in two steps. First, we divided the country in four regions, excluding the three largest Dutch cities, as we knew they could not participate. PCH services were then asked if they could participate in the study. Out of the respondents, we selected nine regional PCH services, stratified by region, that covered both urbanized and non urbanized areas. Second, these PCH services asked parents, invited for a routine well-child visit, to participate. Out of 2426 eligible parents, 2066 were willing to participate (85%); 25 of them provided incomplete data, resulting in a final response of 84%.

PCH professionals were able to provide some information on the non-responders (e.g. age, gender, ethnicity, family composition and past treatment because of psychosocial problems). Responders and non-responders did not differ by age and gender, but children from ethnic minorities were overrepresented among the non-responders (16% vs. 6% among the responders). Table 1 presents background characteristics of the sample and non-responders.

**Table 1:** Characteristics of the respondents and non-respondents

		Response %	Non-response %
Child's gender	male	50	49

	female	50	51

Child's mean age		9.7 (± 1.4)	9.7 (± 1.3)

Ethnic background ^#^	Dutch	83	56

	non-Dutch	6	11

	unknown	10	33

Family composition ^#^	two parents	86	80

	one parent	9	10

	other	5	10

Parental employment	no paid job	3	NA*

	two parents with paid job	53	

	one parent with paid job	34	

	unknown	9	

Parental highest completed education	none or only primary (max. 8 yrs)	3	NA

	lower vocational (max. 12 yrs)	25	

	higher vocational (max 16 yrs)	31	

	university/higher professional (min. 17 yrs)	35	

	unknown	6	

CBCL TPS score in clinical range		9	

Total		n = 2041	

* NA = Not Available

# p < 0.01 (Chi^2^)

### Randomization

Parents received either of the three questionnaires in a random way: the researchers put the CBCL and one of the three other questionnaires in closed envelopes. PCH employees sent these to the parents without knowing which envelopes contained which questionnaires. We found no differences in background characteristics of the sub-samples that received either of the three questionnaires.

#### Determination of Sample Size

We aimed at a sample size of 700 respondents for each of the questionnaires to be evaluated. Earlier studies[1,14] had shown that short questionnaires used in paediatric settings allow for an Area Under the Receiver Operating Curve index (AUC) of about 0.90 with a clinical CBCL Total Problems Score (TPS) as criterion. Sub-sample sizes of 700 are sufficient to detect, with α = 0.05 and a power of 0.80, a difference between AUCs of 10 points around 0.90. PCH services continued data collection until the required number of questionnaires was reached.

### Procedure and Measures

The data were collected during routine preventive health assessments of children aged 7-12, between September 2003 and July 2004. Data collection procedures closely resembled those in other studies [14-16] and were known to result in high response rates. The CBCL and either the SDQ or the PSC or the PSYBOBA were mailed by the PCH services to the parents along with the standard invitation for the well-child assessment, in a closed envelope. Parents filled out the questionnaires, and gave them back to the PCH services in another closed envelope. The services passed them to the researchers, without opening the envelopes.

PCH professionals then interviewed parent and child and examined the child. After each assessment the PCH professionals answered the question: "Does the child have a psychosocial problem?" (yes, no). PCH was explicitly instructed to distinguish between children having psychosocial problems and children that only have risk indicators for such problems. Children for whom only risk indicators but no emotional or behavioural problems were detected, had to be coded as having no psychosocial problems. The phrase 'psychosocial problems' was defined as referring to internalizing and externalizing problems, whether serious or not

The Dutch version of the *CBCL *was used to assess behavioural and emotional problems. The CBCL has a substantive concurrent and predictive validity, also in the Dutch version[5,17-19]. It allows for the calculation of a total problems score (TPS) and several syndrome and broadband scores. Children were allocated to a normal, borderline or clinical range, using the cut-off points recommended in the Dutch manual[18]. These depend on age an gender and are based on the 82^th ^and 90^th ^percentiles of the raw scores in the Dutch normative sample, for a borderline and clinical score, respectively.

The *SDQ Parent Form *is a 33 item behavioural screening questionnaire [11,12,20,21]. It was developed in Great Britain, but is now available in more than 50 languages. Several studies indicate good psychometric properties, in different settings and in different nationalities [22-29]. The SDQ with the Impact supplement consists of 25 items relating to the child's strengths and difficulties and eight items relating to the impact of problems. These last items are not included in the calculation of the total problem scale and were not included in the analysis. The other 25 items allow for the calculation of five subscales (Emotions, Behaviour, Peers, Hyperactivity, Prosocial Behaviour) and a total problem scale (summing the first four sub-scales). We used the parent form of the Dutch version, available at http://WWW.SDQINFO.COM.

The *PSC *is a 35 item questionnaire for parents, assessing psychosocial dysfunction of their children. It was developed in the USA. Several studies have shown its good psychometric properties[9,30-33]. A single total problem score is calculated. No official Dutch version of the PSC was available. Therefore, this questionnaire was translated following a procedure advised by Guillemin, using three independent translators and back-translators and the advice of the original authors [34].

The PSYBOBA is a recently developed Dutch instrument for parents, designed specifically for Dutch PCH [13]. It contains 26 items, on the child's behaviour and emotions. These items allow for the calculation of a single problem scale. Four additional items, on stressful life events and parental worries, are not included in the problem scale. We added three questions to each of the three questionnaires, asking how parents rated length and difficulty and whether they had any remarks on the questionnaires.

Additionally, to get insight in the usability of the questionnaires, nine PCH professionals were asked to use each of the questionnaires in about 25 standard assessments. They rated the usability of the questionnaires, after ten assessments each, in a short rating list. This questionnaire contained ten items on how parents had answered the questionnaires, whether calculation of the scores was considered complicated and so on. Having used each of the three questionnaires PCH professionals answered another questionnaire, in which they rated each questionnaires on a scale from 0 till 10 and indicated which questionnaire they preferred.

The study was approved by the local Medical Ethical Committee, the Commissie Medische Ehiek van het Leiden University Medical Center.

### Analyses

We compared the questionnaires on a number of criteria, developed in studies published before [14,15]. The main criterion variable was a clinical TPS.

First, we assessed the scale structure of the questionnaires. Cronbach's alpha was calculated. We determined the fit between the scale structure and the observed data using Amos 5 Structural Equation Modelling (SEM) [35,36]. We used Structural Equation Modelling (SEM) to assess whether our data fitted the structure of the items in the questionnaires as proposed by their developers. SEM allows to do this by means of a confirmatory factor analysis that answers this question whereas conventional factor analytic methods only allow to do an exploratory factor analysis (i.e. to find the best summary of the information of the various items, without taking into account the structure proposed by the developers). The models tested were considered as fitting when the Parsimony Adjusted Fit Index (PGFI) was higher than 0.90. The models were considered as approximating fitting when the Root Mean Square Error of Approximation (RMSEA) was less than 0.08[36] Items with regression weights ≤ 0.33 were considered as not fitting.

Next, we assessed the validity of the questionnaires, using the TPS as criterion. To assess the construct validity, we calculated the product moment correlation coefficients between the CBCL TPS and the total problem scores on the three short questionnaires. Validity in this context, however, refers primarily to the extent to which the questionnaires can distinguish between children with and without problems. Therefore, also kappa-coefficients were calculated. We calculated the AUCs and tested differences between them by means of bootstrapping. Bootstrapping is a method to estimate the potential chance variation of model parameters on the basis of the empirical distribution of those parameters instead of assumptions on the statistical distribution that these parameters should follow (such as a normal distribution). We drew 1000 samples (with replacements) for each of the three sub-samples of the same sizes as these sub-samples, compared the AUCs for each combination in these samples and calculated the probability that the AUC was smaller or greater. Furthermore, sensitivity and specificity of the three questionnaires were calculated.

The identification of children with problems is of course most relevant for those children who are not yet being treated for such children. We therefore did these analyses not only for the sample as a whole, but also specifically for children who were not treated for any psychosocial problem during the past year (information provided by the PCH professionals).

Originally, we intended to use cut-off points as found in the literature [13,37,38]. However, these cut-off points for the three questionnaires that were compared resulted in considerable differences in the prevalence rates of elevated scores (10% for the SDQ, 5% for the PSC and 32% for the PSYBOBA), while the percentages of children with a clinical CBCL TPS were similar. As a result, the corresponding sensitivity and specificity parameters were very different, whereas the AUCs were quite comparable. We concluded that the sensitivity and specificity based on the original cut-off points were of no use to compare the screening qualities of the three questionnaires. We therefore defined new, more comparable cut-off points, namely those scores that were associated with a specificity of at least 0.90 in our sample. These new cut-off point lead to 16% elevated scores on the PSC and the SDQ and 17% elevated scores on the PSYBOBA.

Third, we assessed the added value of the instruments: to what extend do they offer new information, compared to information on possible risk factors, available from PCH files. These variables are listed in Table 1. In multivariate logistic regression only gender, family composition and ethnic background showed a significant association with a clinical TPS. We performed logistic regression analyses to predict a clinical and borderline TPS, using these variables as predictors. Then we added the elevated scores to the model and checked whether this improved the prediction. This is comparable to what we did in two earlier studies[14,15]. We extended these analyses and also used the question whether the PCH professional had detected any problem (yes or no) as a predictor in the model, before entering the elevated scores. The height of the odds ratio (OR) for elevated scores was used to measure the added value each questionnaire offered.

Finally, the usability of the questionnaire in daily practice was assessed. For parents, differences in response rate, item non-response, and opinions on difficulty and length and the number of critical remarks were tested with ANOVA and Chi2-tests. For PCH professionals' opinions on usability we counted the number of optimal ratings over the individual health assessments for which each questionnaire was used. The comparative ratings of the questionnaires by PCH professionals were inspected and described. No tests were done on these data, as only nine PCH professionals participated in the pilot.

Unless otherwise specified, analyses were done with SPSS 12.

## Results

### Scale Structure

The internal consistencies of the total problem scales of the three questionnaires were very satisfying: 0.80 for the SDQ, 0.87 for the PSYBOBA and 0.89 for the PSC. The differences in Cronbach's alphas could be completely explained by the differences in number of items: applying the Spearman Brown Prophecy Formula resulted in exactly the same corrected α's. Internal consistencies for the SDQ subscales varied between 0.55 and 0.78.

Structural equation modelling showed an unsatisfactory fit of the single scale models with the data (SDQ: PGFI = 0.66; PSC: PGFI = 0.66; PSYBOBA: PGFI = 0.65). RMSEA indices and their 90% confidence intervals were respectively .099 (.094 -104), 079 (.076 -082) and092 (.088 -096), suggesting a mediocre fit for the SDQ and PSYBA and an approximation of fit for the PSC[36]. Table 2 lists the items with low regression weights (≤ .33) in the single scale models. For the SDQ a more subtle model was evaluated, reflecting the questionnaire's subscales. PGFI was 0.74 and RMSEA = .064 (.060 - 0.68). This model may be considered as approximating a fit.

**Table 2:** Items with low regression weights^# ^in the single scale SEM analyses

Questionnaire		Items
SDQ	6	Rather solitary, tends to play alone
	11	Has at least one good friend
	22	Steals from home, school or elsewhere

PSC	1	Complains of aches and pains
	2	Spends more time alone
	3	Tires easily, has little energy.
	15	Less interested in friends
	17	Absent from school
	20	Visits the doctor with doctor finding nothing wrong
	34	Takes things that do not belong to him or her

PSYBOBA	4	Has friends
	7	Dares to stand up for him/her self
	13	Is often still or withdrawn
	14	Often complaints about pain
	15	Is open about what is bothering him/her

# β ≤ 0.33

### Validity

Table 3 presents data on the validity of the three questionnaires, using a clinical and borderline TPS as criterion, for all children and for children who never were treated for any psychosocial problem in the past. Each of the questionnaires was highly correlated with the CBCL TPS. Both the product moment correlation coefficients and the kappa's were quite comparable. The table also presents the adjusted cut-off points used, when calculating kappa, sensitivity and specificity. Differences between the three questionnaires were small. We repeated the analyses for cut-off points which would result in a specificity of at least 0.95. This cut-off point would result in a sensitivity for a clinical TPS of 0.73 for the SDQ, 0.67 for the PSC and 0.62 for the PSYBOBA. Again, no significant differences were found.

**Table 3:** Validity of the questionnaires, using a clinical and borderline CBCL TPS as criteria

	PSYBOBA	PSC	SDQ
Pearson's r	0.81	0.81	0.77

Kappa (cut-off point used)	0.59 (>14)	0.52 (>20)	0.53 (>11)

AUC (95% CI)	0.96 (0.94-0.98)	0.93 (0.92-0.96)	0.95 (0.93-0.98)

**Criterion: Clinical TPS**			

*All children*			

Cut-off point	>14	> 20	> 11

Sensitivity (95% CI)	0.86 (0.78-0.94)	0.78 (0.68-0.88)	0.86 (0.77-0.95)

Specificity (95% CI)	0.91 (0.89-0.93)	0.92(0.89-0.93)	0.90 (0.88-0.92)

Positive Predictive value	0.51 (0.41-0.61)	0.45 (0.35-0.55)	0.43 (0.34-0.52)

*Children currently not under treatment*			

Cut-off point	> 14	>19	>11

Sensitivity (95% CI)	0.82 (0.71-0.93)	0.81 (0.69-0.93)	0.80 (0.67-0.93)

Specificity (95% CI)	0.92 (0.91-0.95)	0.91 (0.89-0.93)	0.92 (0.90-0.94)

Positive Predictive value	0.44 (0.33-0.55)	0.40 (0.30-0.51)	0.37 (0.27-0.48)

**Criterion: Borderline TPS**			

*All children*			

Cut-off point	>13	>18	>10

Sensitivity (95% CI)	0.73 (0.64-0.82)	0.72 (0.64-0.80)	0.79 (0.71-0.87)

Specificity (95% CI)	0.91 (0.89-0.93)	0.91 (0.89-0.93)	0.90 (0.88-0.92)

Positive Predictive value	0.58 (0.49-0.67)	0.62 (0.53-0.70)	0.59 (0.49-0.68)

*Children currently not under treatment*			

Cut-off point	>13	> 17	>10

Sensitivity (95% CI)	0.66 (0.55-0.77)	0.74 (0.65-0.83)	0.75 (0.65-0.85)

Specificity (95% CI)	0.91 (0.89-0.93)	0.91 (0.89-0.93)	0.90 (0.89-0.93)

Positive Predictive value	0.48 (0.38-0.58)	0.57 (0.47-0.66)	0.49 (0.39-0.59)

Excluding children who had been under treatment resulted in almost identical sensitivities and specificities. Using a borderline TPS as the criterion resulted in somewhat lower sensitivities.

### Added Value

Three demographic variables showed a significant association with a clinical TPS: gender, ethnic background and family composition. These variables were included in a logistic regression model as possible predictors of a clinical or borderline/clinical TPS. Adding elevated scores to the model resulted in a significant (p < .001) and substantial improvement of the models for each of the three questionnaires. The Adjusted ORs for all children were: PSYBOBA: 89.3 (38.0 - 210.6); PSC: 35,6 (17.8 - 71.4) and SDQ: 71.7 (30.7 - 167.4). All 95% confidence intervals of the ORs overlapped with each other. For children not under treatment, the ORs were lower but still substantial, ranging from 46.3 till 71.8. The difference, again, were small in relation to the 95% confidence intervals.

Table 4 presents the results of the extended added value analyses, in which problems as detected by the PCH professionals were also included in the model. PCH identified 27% children with a psychosocial problem. The ORs in these extended added value analyses are lower than those in the simple added value analyses, which suggests that part of what is detected by the questionnaires, was also identified by PCH professional without the aid of questionnaires. Yet, in all analyses adding the elevated scores to the model resulted in a very significant (p < .001) improvement of the prediction. The differences between the questionnaires were not significant.

**Table 4:** Odds ratios of the elevated scores on the three questionnaires for a clinical and borderline TPS score, adjusted for gender, ethnic background* and family composition* and Problems identified by PCH**

	PSYBOBA	PSC	SDQ
	**OR (95% CI)**	**OR (95% CI)**	**OR (95% CI)**

**Criterion: Clinical TPS**			

*All children*			

Elevated score yes (versus no)	68.5 (28.3-165.6)	29.3 (14.4-59.8)	55.0 (23.1-131.3)

*Children currently not under treatment*			

Elevated score yes (versus no)	65.4 (24.8-172.4)	40.1 (16.7-96.3)	44.2 (18.0-108.3)

**Criterion: Borderline TPS**			

*All children*			

Elevated score yes (versus no)	22.1 (12.4-39.3)	22.8 (13.3-39.0)	31.3 (16.8-58.6)

*Children currently not under treatment*			

Elevated score yes (versus no)	19.0 (10.1-35.9)	26.8 (14.7-49.0)	29.6 (15.1-58.6)

* see table 1

** Problems identified by PCH professional: yes vs. no

### Usability - Parents

The response rate for the SDQ (87%) was slightly higher than for the PSYBOBA and the PSC (both 84%) (Chi^2 ^= 22.5, df = 2, p < 0.01). The PSC showed the highest mean number of unanswered questions (n = 0.26), compared to 0.06 for the PSYBOBA and 0.05 for the SDQ (F = 7.4, df = 2, 2076, p < 0.001).

Parents' ratings of length, and difficulty of the questionnaires showed significant but small differences between the questionnaires (see Table 5). Twelve percent found the PSYBOBA long or too long, compared to 20% for the PSC and 19% for the SDQ. The percentage of parents rating the questionnaires as difficult varied between 41% (PSC) and 31% (PSYBOBA). Finally, 20% of the parents had criticisms on the PSC, compared to nine percent for the PSYBOBA and ten percent for the SDQ. They criticized the ambiguity of some questions (*e.g*. "Spends more time alone ..." without specification of how to compare) and the discrepancy between items and answering categories (*e.g*. "School grades dropping" to be answered with never, sometimes, or often).

**Table 5:** Parents rating of the length and difficulty of the questionnaires, and percentage of parents with criticisms

	PSYBOBA	PSC	SDQ	Significance^+^
	**n = 796**	**n = 815**	**n = 814**	

Response rate	84%	84%	87%	**

Mean n unanswered items†	0.06	0.18	0.05	***

parents rating: not too long (vs. (too) long)	88%	80%	81%	****

parents rating: (very) easy (vs. (very) difficult, not difficult/not easy)	69%	59%	66%	*

Parents having critical remarks	9%	20%	10%	***

+: p < 0.05; **: p < 0.01; *** p < 0.001

†: Corrected for total number of items in each questionnaire; standardized for 25 items

### Usability - PCH Professionals

PCH professionals rated the three questionnaires on 9 aspects after using them during about 76 examinations. The mean number of optimal ratings was highest for the PSC (6.0 out of 9). For the PSYBOBA it was 5.6 and for the SDQ 4.5 (Table 6). This difference is significant (F = 25.2, df = 2, 241, p < 0.001). The largest differences were found on items referring to the complexity of the calculation of scores, mainly concerning the SDQ's subscales.

**Table 6:** Percentage of examinations resulting in an optimal rating by PCH professionals on nine questions on usability, by questionnaire used

	PSYBOBA	PSC	SDQ
	**%**	**%**	**%**

All items answered	97	88	87

Not irritating at all for the parent	53	57	62

Fully understood	65	62	69

Questionnaire led parents to reflection of the child's mental health	10	8	9

Calculation of scales scores: not difficult	75	98	29

Calculation of scale scores not time consuming	71	88	11

Conversation, based on questionnaire, with parents was useful	11	11	18

Questionnaire covered all parents perceived problems	80	90	87

Questionnaire covered all problems perceived by PCH professional	78	84	78

Mean no of optimal ratings (st. dev)	5.6 (1.6)	6.0 (1.1)	4.5 (1.5)

No of exams	70	81	78

After completing routine examinations with all three questionnaires, PCH professionals rated the questionnaires on a scale from 0 till 10. Mean rating for the SDQ and the PSYBOBA was 6.3, higher than that for the PSC (5.5). When asked which questionnaire they would like to use in the future, four of the eight PCH professionals chose the SDQ, two the PSYBOBA and only one the PSC. One PCH professional did not make a choice.

## Discussion

This study compared three questionnaires (SDQ, PSC and PSYBOBA) in order to decide which was most suited to improve identification of psychosocial problems among children aged 7 till 12 in community health services. The internal consistency of the overall scales was high. The construct validity of the three questionnaires was highly comparable. All questionnaires had a satisfactory sensitivity, at a specificity of 0.90, for problems defined as a clinical TPS score and a somewhat lower sensitivity for problems defined as a borderline TPS score. All three questionnaires offered substantial added value, improving the identification of children with problems based on readily available health indicators and/or clinical assessment during routine examinations. Due to the simplicity of score calculation, the PSC was rated more favourably by nine PCH professionals, for use in daily practice. Yet, in an overall rating by these professionals the PSC was rated less favourably than the SDQ and PSYBOBA.

The PSC resulted in a higher item non response and one in five parents had some criticisms on the PSC. We know no other studies mentioning such problems with the PSC. Yet, we do not think that these problems are to be explained by our translation. Many remarks concerned ambiguities (*e.g*. 'Is less interested in school': less than who or when?) and inconsistencies between the questions and the options for answering ('Gets hurt frequently' to be answered by 'never, sometimes or often). These ambiguities and inconsistencies are also part of the original questionnaire.

An essential element of the design of this study is that it used randomisation and aimed at a comparison of three questionnaires. We know of no other studies that used a similar design. Our approach is comparable to what is now rapidly becoming standard in studies assessing effectiveness and economic evaluations of interventions. Such studies do not assess the effectiveness or costs as such, but compare specific interventions with other interventions or usual care. Such an approach is far more helpful in guiding health policy decisions. We feel that such a comparative approach is worthwhile, too, in the evaluation of questionnaires to be used in health care. Only a systematic comparison can guarantee that the best instrument available will indeed be chosen.

Data were collected using a methodology that closely resembles the way Dutch PCH works, i.e. the questionnaires were sent to the parents, together with the invitation for a regular check up of their child. This improves the external validity of our results. However, one caveat is important: the main aim during sampling was to guarantee similarity between the three sub-samples, not an overall representativeness for the Dutch population. This resulted in a clear underrepresentation of ethnic minorities, caused by a higher nonresponse rate and non-participation of PCH services of the three largest cities in the Netherlands,. The results therefore need confirmation among ethnic minorities.

We used the CBCL as criterion. Although the CBCL is one of the best instruments available and is often used for evaluation purposes, it cannot be regarded as the ultimate golden standard. Other studies used psychiatric interviews or assessments by mental health professionals as standard[8]. Due to financial limitations this was not possible in this study.

The absence of an ultimate golden standard means that the results of the study should be interpreted carefully. The high convergence between the three questionnaires and the CBCL indicates that they can be used as a valuable tool, supporting the professional's assessment and alerting him to probable cases. Yet, it seems unwise to use these short questionnaires as selection tools, limiting further assessment only to those children with elevated scores. The risk of missing serious problems would be too great. The questionnaires are valuable tools to alert PCH professionals on likely cases when they asses psychosocial problems. However, the PCH professional should also include other sources of information: his/her interview with both the parent and the child, the observation of the child and the parent-child interaction, and ratings from teachers whenever possible. Also, the impact of problems on the child's or family's functioning should be taken into account before deciding on further action. The SDQ provides such information in the Impact supplement.

In this study we had to use other cut-off points than those reported in the literature because the latter resulted in large differences regarding the percentage of children with an elevated score. This may raise the question whether these instruments measure the same construct. We think that this is the case indeed, as we found very comparable convergence indices with the CBCL TPS, both for the total scores on each of them and for the proportions with elevated scores based on the adapted cut-off points.

Our data on the validity and reliability of SDQ and the PSC are comparable to those published by other authors[10,11,28-30,37]. We found, however, no other studies which question the scale structures of these questionnaires. Probably, our choice for the rigorous SEM as analytical tool instead of the more usual factor analytical approaches is the key factor. The combination of high internal consistencies and the negative SEM results may come as a surprise. What the SEM analyses showed, however, is that the concepts, as implied by the (sub)scale scores, despite the internal consistencies, are an inadequate description of the way the items are related to each other. In other words: the items provide information not covered by the scale scores. Health care providers should therefore not rely on the scale scores alone, but also carefully check the answers on individual items and discuss these answers with the parents.

Few studies assessed the added value of using questionnaires, as we did. The only studies to compare our results with are our own evaluations of two other questionnaires, that assessed the added value of questionnaires, as compared to risk indicators[14,15]. In the current study we extended the added value analyses, by also including the signals detected by PCH professionals during routine examinations into the analysis. These extended analyses give a better indication of the real added value, as they compare the quality of questionnaire based detection to what is now standard practice for this age group, at least in the Dutch health care system.

Which of the three questionnaires, then, would be the best choice? The sensitivity of the PSC was somewhat less than that for the other questionnaires. The added value of the PSC in detecting children with clinical TPS was relatively low. One in five of the parents had criticisms on the PSC and item non response was also higher. In the Netherlands, the PSC would therefore be an unlikely choice. The psychometric performance of the PSYBOBA and the SDQ were similar. Although more PCH professionals preferred the SDQ as the instrument to use in the future, it was rated less positively in practical use, mainly because by the relative complexity of calculating the (sub)scale scores. We found little support for the supposed scale structure but also found that the SDQ Total Problems score is a strong indicator of problems. When the primary aim is to make a first distinction between children who probably have problems that need attention and those who do not, the single SDQ Total Problems score suffices. This largely simplifies the use of the SDQ.

## Conclusions

This randomized comparison of three questionnaires showed that all three questionnaires can improve the detection of psychosocial dysfunction among children substantially. The PSC elicited more critical remarks from the parents than the SDQ and PSYBOBA and showed a higher item non-response.

## Abbreviations

AUC: Area under Curve; CBCL: Child Behavior Checklist; CI: Confidence Interval;OR: Odds Ratio; PCH: Preventive Child Health Care; PSC: Pediatric Symptom Checklist; PSYBOBA: Psychosociale Problemen Bovenbouw Basisonderwijs (Dutch for: Psychosocial Problems in Primary Education); SDQ: Strengths & Difficulties Questionnaire.

## Competing interests

The authors declare that they have no competing interests.

## Authors' contributions

All authors contributed to the design and execution of the study and the analyses. AGCV, MRC and SAR were actively involved in writing this manuscript. FH commented drafts presented to her.

## Pre-publication history

The pre-publication history for this paper can be accessed here:

http://www.biomedcentral.com/1471-2458/9/489/prepub

## References

[B1] Brugman E Reijneveld SA Verhulst FC Verloove-Vanhorick SP Identification and management of psychosocial problems by preventive child health care Arch Pediatr Adolesc Med 2001 155 462 469 11296073 10.1001/archpedi.155.4.462

[B2] Wildman BG Kinsman AM Logue E Dickey DJ Smucker WD Presentation and management of childhood psychosocial problems J Fam Pract 1997 44 77 84 9010374

[B3] Kelleher KJ McInerny TK Gardner WP Childs GE Wasserman RC Increasing identification of psychosocial problems: 1979-1996 Pediatrics 2000 105 1313 1321 10.1542/peds.105.6.1313 10835074

[B4] Reijneveld SA De Meer G Wiefferink CH Crone MR Parents' concerns about children are highly prevalent but often not confirmed by child doctors and nurses BMC Public Health 2008 8 124 10.1186/1471-2458-8-124 18423036 PMC2383909

[B5] Verhulst FC Ende J Van der Factors associated with child mental health service use in the community J Am Acad Child Adolesc Psychiatry 1997 36 901 909 10.1097/00004583-199707000-00011 9204667

[B6] Durlak JA Wells AM Evaluation of indicated preventive intervention (secondary prevention) mental health programs for children and adolescents Am J Community Psychol 1998 26 775 802 10.1023/A:1022162015815 9861693

[B7] Jellinek M Evans N Knight RB Use of a behavior checklist on a pediatric inpatient unit J Pediatr 1979 94 156 158 10.1016/S0022-3476(79)80386-8 758401

[B8] Jellinek M Murphy JM Robinson J Feins A Lamb S Fenton T Pediatric Symptom Checklist: screening schoolage children for psychosocial dysfunction J Pediatr 1988 112 201 209 10.1016/S0022-3476(88)80056-8 3339501

[B9] Jellinek MS Murphy JM Burns BJ Brief psychosocial screening in outpatient pediatric practice J Pediatr 1986 109 371 378 10.1016/S0022-3476(86)80408-5 3734977

[B10] Jellinek MS Murphy JM Use of the Pediatric Symptom Checklist in outpatient practice Curr Probl Pediatr 1990 602 609 10.1016/0045-9380(90)90031-U

[B11] Goodman R Ford T Simmons H Gatward R Meltzer H Using the Strengths and Difficulties Questionnaire (SDQ) to screen for child psychiatric disorders in a community sample Int Rev Psychiatry 2003 15 166 172 10.1080/0954026021000046128 12745328

[B12] Goodman R Scott S Comparing the Strengths and Difficulties Questionnaire and the Child Behavior Checklist: is small beautiful? J Abnorm Child Psychol 1999 27 17 24 10.1023/A:1022658222914 10197403

[B13] Van Ede J De Rover C Verhappen MJ De Vries-Lequin I PSYBOBA. een pilotstudy naar de mogelijkheden van gebruik van een signaleringsinstrument voor Psychosociale Problematiek in de Bovenbouw van het Basisonderwijs 2003 Utrecht: GGD Nederland

[B14] Reijneveld SA Vogels AG Brugman E van Ede J Verhulst FC Verloove-Vanhorick SP Early detection of psychosocial problems in adolescents: how useful is the Dutch short indicative questionnaire (KIVPA)? Eur J Public Health 2003 13 152 159 10.1093/eurpub/13.2.152 12803414

[B15] Vogels T Reijneveld SA Brugman E Hollander-Gijsman M Verhulst FC Verloove-Vanhorick SP Detecting psychosocial problems among 5-6-year-old children in preventive Child Health Care: the validity of a short questionnaire used in an assessment procedure for detecting psychosocial problems among children Eur J Public Health 2003 13 353 360 10.1093/eurpub/13.4.353 14703324

[B16] Zeijl E Crone MR Wiefferink CH Keizenkamp S Reijneveld SA Children in the Netherlands [in Dutch] 2005 The Hague: SCP

[B17] Bengi-Arslan L Verhulst FC Crijnen AA Prevalence and determinants of minor psychiatric disorder in Turkish immigrants living in The Netherlands Soc Psychiatry Psychiatr Epidemiol 2002 37 118 124 10.1007/s001270200003 11990008

[B18] Verhulst FC Koot HM Manual for the CBCL/4-18 [in Dutch] 1996 Rotterdam: EUR/Sophia Kinderziekenhuis

[B19] Verhulst FC Koot HM Ende J Van der Differential predictive value of parents' and teachers' reports of children's problem behaviors: a longitudinal study J Abnorm Child Psychol 1994 22 531 546 10.1007/BF02168936 7822627

[B20] Bourdon KH Goodman R Rae DS Simpson G Koretz DS The Strengths and Difficulties Questionnaire: U.S. normative data and psychometric properties J Am Acad Child Adolesc Psychiatry 2005 44 557 564 10.1097/01.chi.0000159157.57075.c8 15908838

[B21] Goodman R Meltzer H Bailey V The Strengths and Difficulties Questionnaire: a pilot study on the validity of the self-report version Eur Child Adolesc Psychiatry 1998 7 125 130 10.1007/s007870050057 9826298

[B22] Crone MR Vogels AG Hoekstra F Treffers PD Reijneveld SA A comparison of four scoring methods based on the parent-rated Strengths and Difficulties Questionnaire as used in the Dutch preventive child health care system BMC Public Health 2008 8 106 10.1186/1471-2458-8-106 18394152 PMC2359742

[B23] Goodman R Renfrew D Mullick M Predicting type of psychiatric disorder from Strengths and Difficulties Questionnaire (SDQ) scores in child mental health clinics in London and Dhaka Eur Child Adolesc Psychiatry 2000 9 129 134 10.1007/s007870050008 10926063

[B24] Klasen H Woerner W Rothenberger A Goodman R [German version of the Strength and Difficulties Questionnaire (SDQ-German)--overview and evaluation of initial validation and normative results] Prax Kinderpsychol Kinderpsychiatr 2003 52 491 502 14526759

[B25] Koskelainen M Sourander A Kaljonen A The Strengths and Difficulties Questionnaire among Finnish school-aged children and adolescents Eur Child Adolesc Psychiatry 2000 9 277 284 10.1007/s007870070031 11202103

[B26] Malmberg M Rydell AM Smedje H Validity of the Swedish version of the Strengths and Difficulties Questionnaire (SDQ-Swe) Nord J Psychiatry 2003 57 357 363 10.1080/08039480310002697 14522609

[B27] Mullick MS Goodman R Questionnaire screening for mental health problems in Bangladeshi children: a preliminary study Soc Psychiatry Psychiatr Epidemiol 2001 36 94 99 10.1007/s001270050295 11355451

[B28] Muris P Meesters C Berg F Van den The Strengths and Difficulties Questionnaire (SDQ)--further evidence for its reliability and validity in a community sample of Dutch children and adolescents Eur Child Adolesc Psychiatry 2003 12 1 8 10.1007/s00787-003-0298-2 12601558

[B29] Van Widenfelt BM Goedhart AW Treffers PD Goodman R Dutch version of the Strengths and Difficulties Questionnaire (SDQ) Eur Child Adolesc Psychiatry 2003 12 281 289 10.1007/s00787-003-0341-3 14689260

[B30] Jellinek MS Murphy JM Screening for psychosocial disorders in pediatric practice Am J Dis Child 1988 142 1153 1157 3177320 10.1001/archpedi.1988.02150110031013

[B31] Jellinek MS Murphy JM Little M Pagano ME Comer DM Kelleher KJ Use of the Pediatric Symptom Checklist to screen for psychosocial problems in pediatric primary care: a national feasibility study Arch Pediatr Adolesc Med 1999 153 254 260 10086402 10.1001/archpedi.153.3.254 PMC3905751

[B32] Jutte DP Burgos A Mendoza F Ford CB Huffman LC Use of the Pediatric Symptom Checklist in a low-income, Mexican American population Arch Pediatr Adolesc Med 2003 157 1169 1176 10.1001/archpedi.157.12.1169 14662568

[B33] Reijneveld SA Vogels AG Hoekstra F Crone MR Use of the Pediatric Symptom Checklist for the detection of psychosocial problems in preventive child healthcare BMC Public Health 2006 6 197 10.1186/1471-2458-6-197 16872535 PMC1550396

[B34] Guillemin F Cross-cultural adaptation and validation of health status measures Scand J Rheumatol 1995 24 61 63 10.3109/03009749509099285 7747144

[B35] Arbuckle J Wothke W AMOS 4.0 User Guide 1999 Chicago: Small Waters Corporation

[B36] Byrne BM Structural Equation Modeling with AMOS. Basic Concepts, applications and programnming 2001 Mahwah, New Jersey: Lawrence Erlbaum Associates, Inc. Publishers

[B37] Goedhart A Treffers F Van Widenfelt B Inquiring about mental health problems among children and adolescents: de Strenghts and Difficulties Questionnaire (SDQ) (in Dutch) Maandbl Geestelijke Volksgezondh 2003 58 1018 1035

[B38] Murphy JM Arnett HL Bishop SJ Jellinek MS Reede JY Screening for psychosocial dysfunction in pediatric practice. A naturalistic study of the Pediatric Symptom Checklist Clin Pediatr (Phila) 1992 31 660 667 10.1177/000992289203101104 1424394

